# Gibberellins Play an Essential Role in the Bud Growth of *Petunia hybrida*

**DOI:** 10.3390/cimb46090590

**Published:** 2024-09-05

**Authors:** Jichu Deng, Xinyi Deng, Huanyu Yao, Shunhua Ji, Lili Dong

**Affiliations:** College of Horticulture, Anhui Agricultural University, Hefei 230036, China; 18297978107@163.com (J.D.); xydeng2001@163.com (X.D.); yaohuanyu2666@126.com (H.Y.); jishunhua0325@163.com (S.J.)

**Keywords:** petunia, shoot branching, GA, GID1

## Abstract

This study delves into the role of gibberellin (GA) in governing plant branch development, a process that remains incompletely understood. Through a combination of exogenous hormone treatment, gene expression analysis, and transgenic phenotype investigations, the impact of GA on petunia’s branch development was explored. The results showed that GA_3_ alone did not directly induce axillary bud germination. However, paclobutrazol (PAC), an inhibitor of GA synthesis, effectively inhibited bud growth. Interestingly, the simultaneous application of GA_3_ and 6-BA significantly promoted bud growth in both intact and decapitated plants compared to using 6-BA alone. Moreover, this study observed a significant downregulation of GA synthesis genes, including *GA20ox1*, *GA20ox2*, *GA20ox3, GA3ox1*, and *CPS1*, alongside an upregulation of GA degradation genes such as *GA2ox2*, *GA2ox4*, and *GA2ox8*. The expression of GA signal transduction gene *GID1* and GA response factor *RGA* was found to be upregulated. Notably, the *PhGID1* gene, spanning 1029 bp and encoding 342 amino acids, exhibited higher expression in buds and the lowest expression in leaves. The overexpression of *PhGID1* in Arabidopsis resulted in a noteworthy rise in the number of branches. This study highlights the crucial role of GA in bud germination and growth and the positive regulatory function of GA signaling in shoot branching processes.

## 1. Introduction

Plants are inevitably exposed to complex environmental stresses throughout their lifecycle. Consequently, they have evolved developmental plasticity to cope with adverse conditions. A notable example is the regulation of branching in plants. During this process, axillary meristem forms in the leaf axils and gradually develops into axillary buds, which can remain dormant or activate to form lateral branches [1].

Research conducted over the last few decades has demonstrated that a variety of phytohormones influence branching. In addition to auxin (IAA), cytokinin (CK), and strigolactones (SLs), GA is also involved in regulating branch development, though its mechanism of action is complex. GA plays important roles in many developmental processes, including seed germination, stem elongation, flowering transition, and reproductive development [2]. At present, more than 136 kinds of GAs have been found, but only a very small part of them are biologically active, such as GA_1_, GA_3_, GA_4_, and GA_7_ [3].

The synthesis and metabolic pathways of GA have been comprehensively investigated. The GA biosynthesis process commences with geranylgeranyl diphosphate (GGDP) localized in plastids. This compound is enzymatically transformed by *ent*-copalyl diphosphate synthase (CPS) and ent-kaurene synthase (KS) to yield ent-kaurene, a crucial precursor in the production of GA. Subsequently, ent-kaurene is converted to GA12-aldehyde through the catalytic actions of ent-kaurene oxidase (KO) and ent-kaurenoic acid oxidase (KAO). Ultimately, GA12-aldehyde is metabolized into various GA species by the enzymes GA20ox, GA3ox, and GA2ox [4]. In the signaling pathway, the GA receptor GIBBERELLIN INSENSITIVE DWARF1 (GID1) binds to active GA molecules, transmitting the GA signal to the DELLA protein to form a GID1-GA-DELLA protein complex [5].

GA is frequently considered a branch inhibitor due to observations that GA biosynthesis and GA perception mutants in Arabidopsis, along with GA-deficient transgenic plants in diverse species, typically display branching phenotypes. For instance, the Arabidopsis GA-insensitive mutant, known as *ga*, exhibits characteristics such as reduced apical dominance and increased branching [6]. The GA-deficient mutant *ga1-3* displays more axillary buds [7]. The overexpression of *GA2-oxidase* (*GA2ox*) has resulted in an increased number of tillers or branches in *Oryza sativa* [8], *Solanum lycopersicum* [9], *Paspalum notatum* [10], and *Populus tremula* [11]. Contrary to these findings, several studies have shown that GA stimulates branching. In strawberries, GA biosynthesis mutants have reduced branching, and the application of GA_3_ rescues the phenotype [12]. The application of exogenous GA_3_ to axillary buds can break dormancy in many plants, including woody plants such as *Jatropha curcas* [13], *Prunus avium* [14], and *S. tuberosum* [15], with GA synthesis gene expression levels in buds being positively correlated with bud growth.

GA also regulates branching by interacting with other hormones. Studies have shown that auxin can promote GA synthesis. In pea (*Pisum sativum*), auxin maintains apical dominance by increasing the expression of *GA3-oxidase 1* (*GA3ox*) and inhibiting the expression of *GA2ox1* [16]. When corn tillers are active, endogenous ZR content increases, while GA content decreases, indicating that GA and CK may have antagonistic effects [17]. Numerous studies have shown that GA and SL interact in the growth of lateral buds in various plants. For example, researchers found that D14 and the DELLA protein SLR1 interacted in an SL-dependent manner in *O. sativa*, suggesting an interaction between the SL and GA signaling pathways [18]. In *Malus pumila*, GA_3_ treatment significantly affected the expression of *MdMAX1*, *MdD14*, and *MdMAX2* [19]. These studies indicate that GA regulates branch development through interactions with auxin, CK, and SL.

Although the role of GA in regulating branch development has been studied in multiple species and significant progress has been made, the mechanism of action of GA is still unclear, which requires us to search for more experimental evidence in more species. *P. hybrida*, originally from South America, has now spread all over the world and is known as the king of herbaceous flowers. The number of branches determines the quantity of petunia flowers, thereby affecting their ornamental characteristics. Therefore, the regulation of branch number is an important research direction in *P. hybrida*. In this study, the role of GA in regulating branching was preliminarily investigated through hormone application, gene expression analysis, and a functional analysis of GA pathway-related genes. This research lays a foundation for understanding the mechanism of branch development regulation in petunia and provides an experimental basis for the future application of hormones in agriculture.

## 2. Materials and Methods

### 2.1. Plant Growth Conditions

The petunia cultivar employed in our research was Petunia × hybrida cv ‘Mitchell Diploid’. Both petunia and Arabidopsis Columbia (Col-0) plants, as well as transgenic plants, were cultivated in tissue culture rooms under the following growth conditions: temperature 23 ± 2 °C, photoperiod of 16 h/8 h (light/dark), and light intensity ranging from 3500 to 4000 LX.

### 2.2. Hormonal Application

Forty-five-day-old petunia plants were selected, and 10 µL of hormone solution was applied to the axillary bud using a pipette. The treatments included GA_3_, 6-BA+GA_3_, PAC, and 6-BA. Fifteen plants were used for each treatment. Bud length was measured and photographed every three days. This experiment was repeated three times to ensure accuracy. The hormone concentrations used in this experiment are shown in Supplemental Table S1.

### 2.3. Isolation of Genes

According to the published petunia genome sequence, the full-length cDNA of *PhGID1*, *PhBRC1*, and *PhDRM1* was obtained using primers specified in Table 1. The amplified products, produced with MegaFi^TM^ Fidelity 2 × PCR MasterMix (Abm, Cologne, Germany), were sequenced by General Biosystems (Anhui) Company in Chuzhou, China.

The GID1 sequences were all downloaded from NCBI (https://www.ncbi.nlm.nih.gov/, accessed on 2 January 2023). In the process of constructing the phylogenetic tree, MEGA11 software was utilized. The neighbor-joining (NJ) method was applied, accompanied by 1000 bootstrap replicates for enhanced reliability [20]. The alignment of multiple sequences was carried out using DNAMAN 7.0 software. In addition, the physicochemical characteristic analysis of PhGID1 was carried out using ProtParamtool (https://web.expasy.org/protparam/, accessed on 2 January 2023) [21].

### 2.4. Analysis of Gene Expression

For the tissue expression experiment, flowers, buds, leaves, stems, and roots of 90-day-old petunia plants were collected. For the hormone treatment experiment, buds subjected to different treatments were collected. RNA extraction was carried out using the PastPure^®^ Universal Plant Total RNA Isolation Kit (Vazyme, Nanjing, China). In RT-PCRs, two micrograms of total RNA was utilized alongside MonScript^TM^ RTIII Super Mix, which incorporates dsDNase (Two-Step) (Monad, Suzhou, China). qRT-PCR was performed following the protocol of the SYBER premix ExTaq kit (TaKaRa, Dalian, China) on an Applied Biosystems Plus Real-Time PCR System. *PhGAPDH* expression served as the normalization reference. Primers used for the qRT-PCR were designed utilizing Primer Premier 5 software (PREMIER Biosoft Int., Palo Alto, CA, USA) (Table 1). Three technical replicates and three biological replicates were performed for every sample. The target genes’ relative expression levels were verified based on the 2^−ΔΔCt^ method [22].

### 2.5. Arabidopsis Transformation and Phenotype Analysis

Primers PhGID1-1300-F and PhGID1-1300-R were utilized to amplify *PhGID1*. The resulting PCR products and pSuper1300 vector were both digested with *Hind*III and *Sal*I. The recombinant reaction was carried out following the guidelines of the NovoRec^®^ plus One-step PCR Cloning Kit (Novoprotein, Shanghai, China). Subsequently, the recombinant product was transformed into competent cells of *Escherichia coli* DH5α. Upon screening for positive clones, the plasmid of pSuper1300-PhGID1 was isolated and transformed into an *Agrobacterium tumefaciens* GV3101 strain using the freeze/thaw method.

The constructed 35S::PhGID1 was delivered into Arabidopsis plants (Col-0) utilizing the floral-dipping technique [23] with the Agrobacterium strain. Seeds harvested from the injected plants were cultured on Murashige and Skoog (MS) medium supplemented with 50 mg·L^−1^ kanamycin. After 15 days of germination, seedlings resistant to kanamycin were transplanted to soil and placed in a growth chamber. To conduct the phenotypic analysis, the height of the main stems and the count of branches (bud length ≥10 mm) were evaluated.

### 2.6. Data Analysis

The bar chart and line chart data in this experiment were calculated using Excel 2016 software and are expressed as the mean ± standard deviation. Meanwhile, significance analysis was performed on the data. A *p*-value less than 0.05 (* *p* < 0.05, ** *p* < 0.01, *** *p* < 0.001) indicated a significant difference.

## 3. Results

### 3.1. Exogenous GA_3_ Could Not Promote Axillary Bud Germination in Petunia

**Figure 1:**
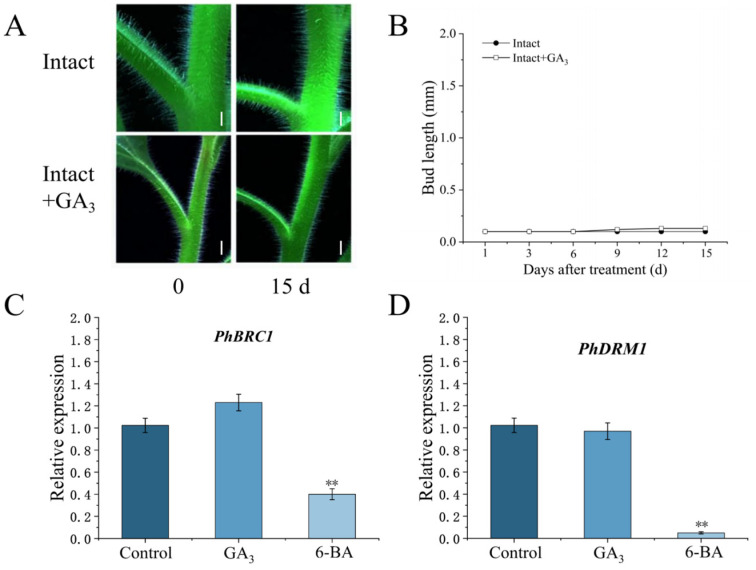
Exogenous GA_3_ cannot promote bud germination in petunia. (**A**) Axillary bud growth of petunia plants after GA_3_ treatment, bars = 1 cm. (**B**) Statistical analysis of bud length of petunia. (**C**,**D**) Regulation of GA_3_ and 6-BA on *PhBRC1* and *PhDRM1* expression. (** *p* < 0.01).

To investigate the effect of GA on bud germination in petunia, exogenous GA_3_ was applied to the fourth axillary bud from top to bottom, and the growth of the axillary bud was recorded over 15 days. As shown in Figure 1, the application of GA_3_ to buds did not promote bud germination.

Furthermore, we sampled the leaf axils treated with GA_3_ and measured the expression levels of *PhBRC1* and *PhDRM1*, which are key genes in bud dormancy [24,25]. The results showed that after six hours of 6-BA treatment, *PhBRC1* and *PhDRM1* were downregulated to 0.42 and 0.09 times the control levels, respectively. However, GA_3_ treatment did not cause meaningful changes in the expression levels of *PhDRM1* and *PhBRC1* (Figure 1). These findings indicated that exogenous GA_3_ could not activate bud germination in petunia.

### 3.2. GA Promotes Bud Elongation

**Figure 2:**
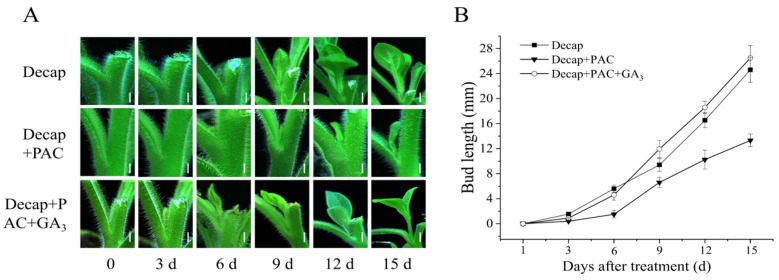
GA promoted bud elongation in petunia. (**A**) Bud growth of petunia after different treatments, bars = 1 cm. (**B**) Statistical analysis of bud length of petunia.

Since GA cannot promote the germination of axillary buds, we aimed to determine whether GA is involved in the growth of axillary buds after germination. PAC and PAC+GA_3_ were applied to the buds after decapitation. As shown in Figure 2, on day 15, the axillary buds of the decapitation (Decap) group reached 24.6 mm, the bud length in the Decap+PAC group was 13.32 mm, and the bud length in the Decap+PAC+GA_3_ group was 26.5 mm. These results indicated that PAC significantly inhibited bud elongation after decapitation but did not affect bud germination. The application of GA_3_ counteracted the inhibitory effect of PAC on bud elongation, implying that the inhibition of axillary bud growth is indeed caused by the deficiency of endogenous GA_3_.

### 3.3. GA and CK Synergistically Promote Bud Elongation

**Figure 3:**
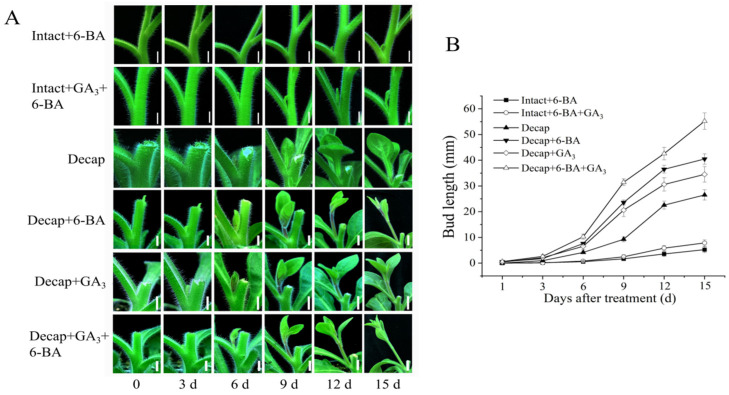
GA and CK synergistically promoted bud growth in petunia. (**A**) Bud growth of petunia with different treatments, bars = 1 cm. (**B**) Statistical analysis of bud length of petunia.

To study the interaction between GA and CK in regulating bud elongation, we treated the buds with various solutions. As shown in Figure 3, on day 15, the bud length for the Intact+6-BA, Intact+GA_3_+6-BA, Decap, Decap+6-BA, Decap+GA_3_, and Decap+GA_3_+6-BA groups were 5.23 mm, 7.84 mm, 28.5 mm, 40.5 mm, 34.5 mm, and 55.2 mm, respectively. These results indicated that the simultaneous application of GA_3_ and 6-BA could significantly promote bud elongation in both intact plants and decapitated plants, compared to the single application of GA_3_ and 6-BA. This suggests that GA and CK have a synergistic effect in regulating the bud growth of petunia.

### 3.4. GA Pathway Genes Respond to Decapitation

**Figure 4:**
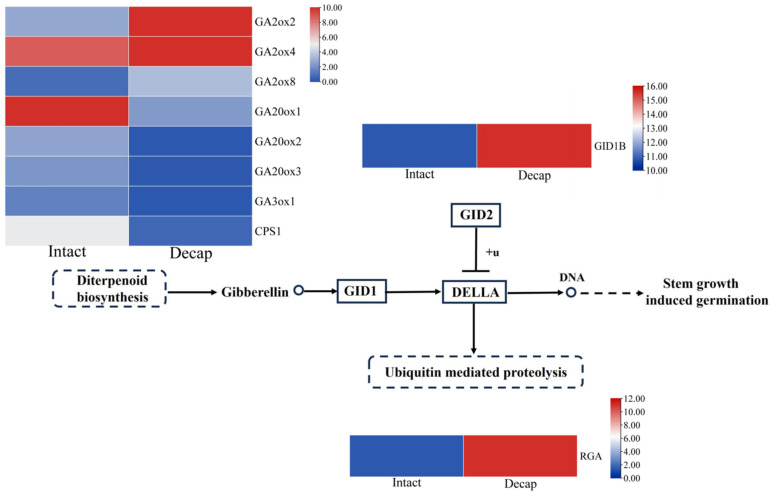
Expression analysis of GA pathway genes after decapitation for 1 h.

Given that exogenous GA cannot directly promote the germination of axillary buds in petunia, we investigated whether endogenous GA plays a crucial role in bud germination. To explore this issue, we measured the expression levels of GA pathway genes after decapitation. As depicted in Figure 4, 1 h after decapitation, the expression levels of bioactive GA-degrading enzyme genes *GA2ox2*, *GA2ox4*, and *GA2ox8* were upregulated by 5.44, 3.86, and 4.06 times compared to the control, respectively. The expression levels of GA synthetic genes *GA20ox1*, *GA20ox2*, *GA20ox3*, and *GA3ox1* and the first key enzyme of the GA biosynthetic pathway *CPS1* decreased to 0.05, 0.05, 0.07, 0.09, and 0.13 times the control levels, respectively. Moreover, the expression of the GA signal transduction gene *GID1B* was upregulated from 10.8 to 15.6, and the *REPRESSOR OF ga1-3* (*RGA*), a repressor of GA signaling, was upregulated by 7.86 times compared to the control. These results suggest that these GA pathway genes may be involved in bud activation.

### 3.5. Cloning of PhGID1 and Sequence Analysis

**Figure 5:**
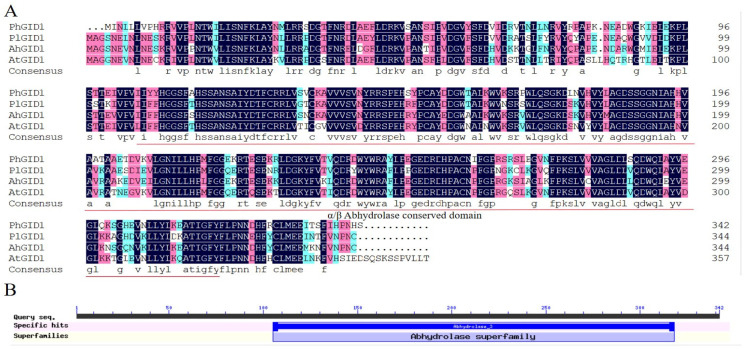
Sequence analysis of PhGID1. (**A**) Comparison of GID1 amino acid sequences of various plants. Note: Pl: *P. lactiflora*; Ah: *A. hypogaea*; At: *A. thaliana.* (**B**) Conserved domain analysis of PhGID1.

Considering the well-documented branching phenotype of GA synthesis genes in transgenic plants and the relatively limited research on the involvement of the GA signal transduction gene *GID1* in branch development, we selected this gene for further study. The *PhGID1* sequence is 1029 bp long and encodes 342 amino acids. The molecular formula of the PhGID1 protein was estimated to be C_1756_H_2693_N_483_O_501_S_9_. The amino acid composition of the protein includes 9.9% Leu, 9.1% Val, 7.6% Ser, 6.4% Ala, and 6.4% Arg. The total number of negatively charged residues (Asp+Glu) is 39, while the total number of positively charged residues (Arg+Lys) is 38. Comparative sequence analysis revealed that the protein sequences of PhGID1 share similarities of 78.26% with *Paeonia lactiflora*, 77.10% with *Arachis hypogaea*, and 72.91% with *A. thaliana*, (Figure 5A). Additionally, sequence analysis showed that PhGID1 contains an α/β hydrolase-conserved domain (Figure 5B).

### 3.6. Analysis of Tissue-Specific Expression of PhGID1

**Figure 6:**
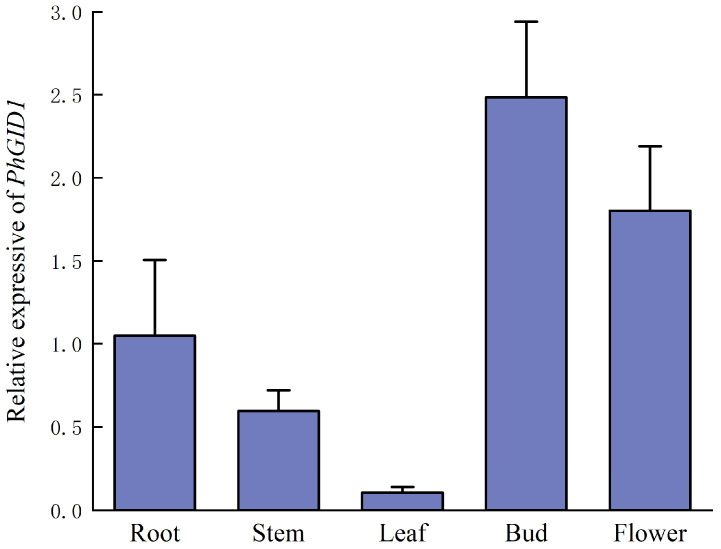
The relative expression of *PhGID1* in various tissues of petunia.

To investigate the expression characteristics of *PhGID1*, we measured its expression levels in various tissues, including the roots, stems, leaves, buds, and flowers of petunia (Figure 6). The results revealed that *PhGID1* is expressed in all examined tissues, with the highest expression levels observed in buds and the lowest in leaves. The expression levels of *PhGID1* in the roots, stems, leaves, and flowers were 0.42, 0.24, 0.04, and 0.72 times that in buds, respectively.

### 3.7. Overexpression of PhGID1 Increases Branch Number

**Figure 7:**
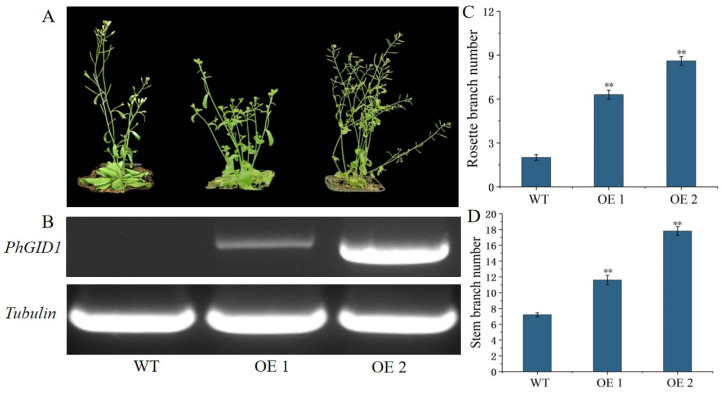
Phenotypic analysis of transgenic plants overexpressing *PhGID1*. (**A**) Phenotypic comparisons of WT and transgenic plants overexpressing *PhGID1*. Different lines overexpressing *PhGID1* are represented by OE 1 and OE 2. (**B**) RT-PCR evaluated expression levels of *PhGID1*. (**C**) Statistics of rosette branch number (*n* = 15). (**D**) Statistics of stem branch number (*n* = 15). (** *p* < 0.01).

To elucidate the function of *PhGID1*, we constructed the *35S::PhGID1* vector and transformed it into Arabidopsis, resulting in nine transgenic lines. Two transgenic lines (OE 1 and OE 2) were selected for phenotypic analysis. As shown in Figure 7, the overexpression of *PhGID1* led to a significant increase in the number of branches. Compared with control plants, the average number of rosette branches in OE 1 and OE 2 was 5.2 and 6.5, respectively, while the average number of stem branches was 11.4 and 16.8, respectively.

## 4. Discussion

The branching stage of plants includes the establishment of axillary meristem (AM), the formation and germination of axillary buds, and the elongation of buds. Each stage is regulated by a variety of genes and hormones [26]. Although many studies have reported on the regulation of branch development by GA, their findings are inconsistent, making the role of GA in this process unclear.

In our study, we revealed partial functions of GA through hormone application and transgenic experiments. Directly applying GA_3_ to the axillary buds of petunia did not promote their germination, nor did it alter the expression levels of *PhBRC1* and *PhDRM1*. This finding is consistent with studies in pea and *M. pumila* [19,27], but it is contrary to research on *J*. *curcas* and sweet cherry [13,14]. These differences may be related to the state of axillary buds treated, rather than species-specific responses to GA. Both our experiment and Cao et al.’s study found that GA can promote the growth of axillary buds when they are in an activated state.

Even though GA cannot directly stimulate bud germination like CK can, the application of GA_3_ following decapitation can promote bud elongation, which aligns with the finding that GA promotes the growth of buds released by decapitation in peas [27]. Applying PAC to axillary buds after decapitation significantly inhibited their growth but did not affect their germination, indicating that GA’s role in bud germination is not necessary. The combination of PAC+GA_3_ restored PAC’s inhibition of bud growth, indicating that GA is crucial in regulating bud elongation.

Simultaneously applying GA_3_ and 6-BA to axillary buds significantly promoted bud elongation, both after decapitation and in intact plants. This phenomenon was observed in both pea and *M. pumila*, suggesting that the synergistic effect of GA and CK in promoting axillary bud elongation is widespread. Studies on *J*. *curcas* have shown that this regulation by GA and CK is due to CK promoting the biosynthesis of GA in *J*. *curcas* buds and inhibiting GA degradation [13]. Further research is needed to determine whether other mechanisms underlie this synergistic effect.

To examine whether GA is involved in the activation of axillary buds, we performed a heatmap analysis on the expression levels of GA pathway genes 1 h after decapitation. The results showed that the synthesis genes of GA (*GA20ox1*, *GA20ox2*, *GA20ox3*, *GA3ox1*, and *CPS1*) were significantly downregulated, while the degradation genes (*GA2ox2*, *GA2ox4*, and *GA2ox8*) were significantly upregulated. Earlier studies have revealed that rising GA levels in leaf axils through the ectopic expression of the GA biosynthesis enzyme *GA20ox2* markedly impaired axillary meristem initiation [7]. These investigations indicate that low GA content plays a vital role in the initiation of the axillary meristem and the activation of axillary buds.

In the transcriptome data, we observed an upregulation of *PhGID1B* expression levels, though not significantly, and did not detect other members of *GID1*. Despite the modest upregulation, *PhGID1B* expression levels were higher than most other genes both before and after decapitation. Combined with tissue-specific expression experiments, *PhGID1B* showed the highest expression level in axillary buds, suggesting its potential involvement in regulating branch development in petunia. However, there is limited research on the role of *GID1B* in branch development. Therefore, we cloned this gene and analyzed its expression in a variety of tissues. We found that this gene was highly expressed in buds and had the lowest expression in leaves. This contrasts with results in *P. tomentosa*, where higher expression was observed in leaves compared to roots [28]. To clarify the function of this gene, we constructed the overexpression vector 35S::PhGID1B and transformed it into Arabidopsis. The overexpression of *PhGID1B* resulted in changes in both rosette and stem branches in Arabidopsis, implying that *PhGID1B* is involved in regulating branch development.

Based on the above research, we propose that GA plays a critical role in the activation and elongation of axillary buds. Low levels of GA are beneficial for the activation of axillary buds, while high levels of GA contribute to the growth of buds. Therefore, the synthesis of GA may be dynamic throughout the entire process from the formation of the AM to the development of lateral branches, exhibiting different levels at various stages. For the GA signal transduction gene, although there was no significant change in expression level during the activation of buds after decapitation, it still played a role in bud germination, as evidenced by the increased number of branches in transgenic plants overexpressing *PhGID1B*. This research provides a theoretical reference for enriching the regulatory mechanism of GA in branch development.

## Figures and Tables

**Table 1:** Primer sequence used in PCR and qRT-PCR.

Primer Name	Primer Sequence (5′ to 3′)
PhGID1-F	ATGATCAATTTGCTTTTAGTACC
PhGID1-R	CTATGAATGGTTAGGATGGATGA
qRT-PhGID1-F	CGACGGTCACCTGAACATAG
qRT-PhGID1-R	TTCTCGGATTCGGTCCTTTT
PhGID1-1300-F	CCAAATCGACTCTAGAATGATCAATTTGCTTTTAGTACC
PhGID1-1300-R	CCACTAGTATTTAAATGCTATGAATGGTTAGGATGGATGA
PhBRC1-F	CCCATTTGCTCATCTTTATTC
PhBRC1-R	CTGCCACTTTGCTTACTCATA
PhDRM1-F	GAATGTGTGGAGGAGTGTTTTT
PhDRM1-R	CCATATTGCACCCCCTTTTGTT

## Data Availability

Enquiries about data availability should be directed to the authors.

## Supplementary Materials

The following supporting information can be downloaded at https://www.mdpi.com/article/10.3390/cimb46090590/s1. Table S1: Hormone concentrations.
