# Supplementary material for: Gibberellins Play an Essential Role in the Bud Growth of Petunia hybrida
Source: Curr Issues Mol Biol. 2024 Sep 5;46(9):9906–15. doi: 10.3390/cimb46090590 (PMC11430761; doi:10.3390/cimb46090590)
Supplement: Supplementary file 1 [file cimb-46-00590-s001.zip › cimb-3162545-supplementary.pdf]

Table S1. Hormone concentrations

Treatments	100 mM PAC (in DMSO)(μL)	100 mM GA3 (in DMSO) (μL)	200 mM 6-BA (in DMSO)(μL)	DMSO (μL)	Tween 20 (μL)	Water (μL)	Total Volume (μL)
PAC	1	0	0	0	10	989	1
6-BA	0	0	0.5	0.5	10	989	1
GA_3	0	1	0	0	10	989	1
GA_3+6BA	0	0.5	0.5	0	10	989	1
Control	0	0	0	1 μl	10	989	1
